# Lack of independent effect of type 2 diabetes beyond characteristic comorbidities and medications on small muscle mass exercising muscle blood flow and exercise tolerance

**DOI:** 10.14814/phy2.12487

**Published:** 2015-08-11

**Authors:** Veronica J Poitras, Robert F Bentley, Diana H Hopkins-Rosseel, Stephen A LaHaye, Michael E Tschakovsky

**Affiliations:** 1 School of Kinesiology and Health Studies, Queen’s UniversityKingston, Ontario, Canada; 2 Cardiac Rehabilitation Centre, Hotel Dieu HospitalKingston, Ontario, Canada; 3 School of Rehabilitation Therapy, Queen’s UniversityKingston, Ontario, Canada

**Keywords:** Critical power, exercise hyperemia, oxygen delivery, skeletal muscle perfusion, ultrasound, vascular function

## Abstract

Persons with type 2 diabetes (T2D) are believed to have reduced exercise tolerance; this may be partly due to impaired exercising muscle blood flow (MBF). Whether there is an impact of T2D on exercising MBF within the typical constellation of comorbidities (hypertension, dyslipidemia, obesity) and their associated medications has not been investigated. We tested the hypothesis that small muscle mass exercise tolerance is reduced in persons with T2D versus Controls (matched for age, body mass index, fitness, comorbidities, non-T2D medications) and that this is related to blunted MBF. Eight persons with T2D and eight controls completed a forearm critical force (fCF_impulse_) test as a measure of exercise tolerance (10-min intermittent maximal effort forearm contractions; the average contraction impulse in the last 30 sec quantified fCF_impulse_). Forearm blood flow (FBF; ultrasound) and mean arterial pressure (MAP; finger photoplethysmography) were measured; forearm vascular conductance (FVK) was calculated. Data are means ± SD, T2D versus Control. fCF_impulse_ was not different between groups (136.9 ± 47.3  N·sec vs. 163.1 ± 49.7 N·sec, *P *= 0.371) nor was the ΔFBF from rest to during exercise at fCF_impulse_ (502.9 ± 144.6 vs. 709.1 ± 289.2 mL/min, *P *= 0.092), or its determinants ΔFVK and ΔMAP (both *P *> 0.05), although there was considerable interindividual variability. ΔFBF was strongly related to fCF_impulse_ (*r *= 0.727, *P *= 0.002), providing support for the relationship between oxygen delivery and exercise tolerance. We conclude that small muscle mass exercising MBF and exercise tolerance are not impaired in representative persons with T2D versus appropriately matched controls. This suggests that peripheral vascular control impairment does not contribute to reduced exercise tolerance in this population.

## Introduction

It is often stated that persons with type 2 diabetes (T2D) have exaggerated intolerance to exercise beyond what might be explained by sedentary behavior [for review, see Reusch et al. 2013) and that this contributes to poor adherence to physical activity, which is an important treatment modality (Stewart 2002; Sigal et al. 2004). Advocates for this view cite observations of slowed oxygen uptake kinetics and reduced peak oxygen consumption capacity (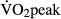) relative to (self-reported) activity-matched controls (Regensteiner et al. 1995, 1998; Baldi et al. 2003; Lalande et al. 2008). Importantly, however, evidence of reduced exercise tolerance when matched for 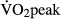 is absent from the literature, and since 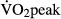 (kinetics and peak) responds normally to exercise training in this population (Reusch et al. 2013) it is unclear how much of the “impairment” in tolerance is due to relative inactivity versus deficiency that is inherent to the disease. While a single study reported that ratings of perceived exertion were greater after adjusting for relative work intensity in persons with T2D versus weight-matched controls (*n = *13 per group), this was only true at a low-level work rate (∼3 metabolic equivalents of task; METs), these ratings are subjective, and the underlying physiological mechanism was not elucidated (Huebschmann et al. 2009).

If, in fact, exaggerated exercise intolerance is present in persons with T2D, it has been proposed that reduced exercising muscle blood flow and concomitant impaired oxygen delivery, factors which are known to increase fatigue progression (Amann et al. 2006; Katayama et al. 2007), are at least partly responsible (Kingwell et al. 2003; Lalande et al. 2008). T2D is characterized by vascular pathologies (Hogikyan et al. 1999; James et al. 2004; Sprague et al. 2006; Montero et al. 2013) which could impact vascular responsiveness in exercising muscle, blunting oxygen delivery and exercise tolerance. Previous studies have investigated exercising muscle oxygen delivery either in persons with T2D who are comorbidity- and medication-free compared to weight- and age-matched controls (Bauer et al. 2007; Lalande et al. 2008) or who had medications discontinued during the study (Martin et al. 1995; Kingwell et al. 2003; Womack et al. 2009), or did not have control groups matched for comorbidities and their related medications (Womack et al. 2009; Joshi et al. 2010; MacAnaney et al. 2011; Thaning et al. 2011). However, persons with T2D typically also present with obesity (Sharma and Jain 2009), hypertension (Savoia and Schiffrin 2007), and/or dyslipidemia (Chehade et al. 2013) and are taking one or more associated medications, as well as medications for T2D, and as such findings from previous studies do not have ecological validity for this population.

Whether there is an impact of T2D on exercising muscle blood flow within the characteristic constellation of comorbidities and medications has not been investigated. In addition, if impaired muscle blood flow is present in this population, the extent to which it impacts exercise tolerance is unknown. Reduced exercising muscle blood flow could be due to impaired peripheral vascular control (i.e., reduced vasodilation), but it could also be due to impaired cardiac function which might evoke an increase in sympathetic vasoconstriction in exercising muscle (Zhang et al. 1985; Saltin and Strange 1992), and thus investigation of peripheral vascular dysfunction specifically requires the use of a small muscle mass modality that would be unlikely to be limited by impaired cardiac function. Therefore, the aim of this study was to assess small muscle mass exercising muscle blood flow and exercise tolerance in persons with T2D versus Control participants matched for age, body mass index (BMI), aerobic fitness, comorbidities, and non-T2D medications. We hypothesized that the T2D group would have reduced exercising muscle blood flow and that this would be associated with reduced exercise tolerance. If present, reduced muscle blood flow and associated impaired small muscle mass exercise tolerance would be one factor contributing to whole-body exercise intolerance in this population.

We addressed the limitations of the current literature by: (1) matching T2D and control participants for peak aerobic capacity as estimated by peak METs achieved in a graded treadmill exercise test, and for other potentially confounding factors including BMI and medication use, (2) utilizing a small muscle mass exercise modality that enables isolation of peripheral vascular contributions to exercising muscle blood flow (Saltin and Strange 1992; Secher and Volianitis 2006), and (3) using a novel test of small muscle mass exercise tolerance that has been shown to be sensitive to differences in oxygen delivery: the forearm critical force (fCF_impulse_) test (Kellawan and Tschakovsky 2014).

## Methods

### Participants

Ten men with T2D and 10 matched controls participated in this study. Participants were recruited via the Cardiac Rehabilitation Centre at Hotel Dieu Hospital (HDH, Kingston, ON), and all testing was completed in the interim between their screening for, and commencement of, this program. The important rationale for this approach to recruitment was to enable investigation of the impact of T2D within the constellation of prevalent comorbidities and medications and to match control participants for these characteristics in order to isolate an effect of T2D.

**Figure 1:**
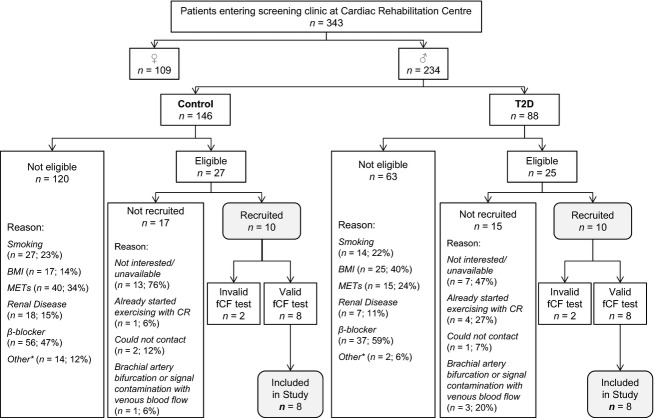
Flowchart of participant recruitment. Note that potential participants may have been deemed ineligible due to one or more criteria (therefore the *n* values for the reasons participants were “not eligible” do not sum to the total number of participants who were not eligible). *Other reasons for exclusion were: myocardial ischemia during exercise (*n* = 1), Churg–Strauss syndrome (autoimmune vasculitis; *n* = 1), heart transplant patient (*n* = 1), age (*n* = 2; 17 and 85 years), unable to do baseline assessment of aerobic fitness (*n* = 3; due to: wheelchair, high blood pressure, musculoskeletal issues), participation vetoed by cardiologist without provided reason (*n* = 2), impaired glucose tolerance (*n* = 6). BMI, body mass index; CR, cardiac rehabilitation; fCF, forearm critical force; METs, metabolic equivalents.

Inclusion criteria were the following: (1) BMI of 24–35 kg/m^2^, (2) achievement of 4–10.0 METs on a symptom-limited graded treadmill exercise test, (3) clear presence or absence of T2D [*T2D*: fasting plasma glucose ≥7.0 mmol/L *and/or* oral glucose tolerance test (OGGT) 2 h plasma glucose ≥11.1 mmol/L; *control*: fasting plasma glucose <6.1 mmol/L *and* OGGT 2 h plasma glucose <7.8 mmol/L], and (4) successful completion of the forearm critical force test (see “Experimental protocol” below). Participants were excluded from the study if they demonstrated: (1) Stage 3 (or more advanced) renal disease, (2) current smoking or smoking within the past 12 months, or (3) if they were taking exclusionary medications (*β*-blockers, nitroglycerine, or other nitric oxide donors). Participants taking *β*-blockers were not excluded if they could safely withdraw these medications for 48 h prior to testing, under medical supervision (Dr. Stephen LaHaye, Cardiologist at HDH) (*n = *1 in each group). Participants completed testing while continuing any other medications (Table1). See Figure1 for a flowchart of participant recruitment.

**Table 1:** Participant characteristics

Variable	Control group	T2D group	*P* value
Age, years	62.6 ± 10.7	61.8 ± 8.9	0.862
Diabetes duration, years	–	6.9 ± 6.2	–
Height, cm	180.0 ± 6.6	175.1 ± 5.3	0.122
Weight, kg	96.0 ± 19.6	97.7 ± 16.4	0.861
BMI, kg/m^2^	29.5 ± 4.5	31.8 ± 4.4	0.328
WC, cm	104.6 ± 9.8	109.8 ± 12.6	0.372
Fasting plasma glucose, mmol/L	5.1 ± 0.4	8.1 ± 2.0	0.001*
HbA1c, %	5.6 ± 0.4	7.3 ± 1.6	0.009*
Fasting triglycerides, mmol/L	1.9 ± 2.6	1.6 ± 1.1	0.767
Total cholesterol, mmol/L	3.3 ± 1.3	3.4 ± 1.1	0.797
HDL cholesterol, mmol/L	1.1 ± 0.3	1.0 ± 0.2	0.787
LDL cholesterol, mmol/L	1.3 ± 0.4	1.7 ± 0.7	0.270
Baseline GXT, METs	8.4 ± 1.0	8.2 ± 1.7	0.777
Forearm volume, mL	1269.6 ± 260.1	1267.5 ± 216.4	0.986
Forearm circumference, cm	29.0 ± 2.5	29.9 ± 3.2	0.532
Arm MVC, kg	37.5 ± 9.3	35.9 ± 10.3	0.737
Number of non-T2D medications	4.5 ± 1.1	4.8 ± 1.9	0.751

	Number (*n*) using medications		
COX inhibitor (aspirin)	6	7	–
ACE inhibitor	6	8	–
Angiotensin II receptor blocker	2	0	–
HMG-CoA reductase inhibitor (“statin”)	7	8	–
Thienopyridine (or derivative; “antiplatelet”)	3	3	–
Cholesterol absorption inhibitor	0	1	–
Biguanide	0	7	–
Insulin/Analog	0	1	–
Other	7	4	–

	Cardiovascular health history (*n*)		
Acute myocardial infarction	2	2	–
Angioplasty	4	5	–
Coronary artery bypass surgery	5	2	–
History of cardiomyopathy	0	1	–
Heart valve repair/replacement	0	3	–
Transient ischemic stroke	1	0	–

Values are means ± SD.

ACE, angiotensin-converting enzyme; BMI, body mass index; COX, cyclooxygenase; GXT, graded exercise test (treadmill); HDL, high-density lipoprotein; HMG-CoA reductase, 3-hydroxy-3-methyl-glutaryl-coA reductase; LDL, low-density lipoprotein; METs, metabolic equivalents; MVC, maximum voluntary contraction; WC, waist circumference.

* *P* < 0.05 compared with control group.

Prior to the day of testing, participants visited the laboratory to be screened for clear blood velocity signals (Doppler ultrasound) and images (Echo ultrasound) of the brachial artery, and to perform a forearm critical force test (fCF_impulse_; described below) for purposes of familiarization (Kellawan and Tschakovsky 2014). The study protocol was approved by the Health Sciences Human Research Ethics Board at Queen’s University, and participants gave written consent to participate on forms approved by this board.

### Experimental protocol

Participants were instructed to abstain from taking anti-inflammatories within 48 h of the study (excluding daily low-dose Aspirin; *n = *7 in T2D group, *n = *6 in control group), from exercising within 24 h, and from consuming alcohol or caffeine for 12 h and food for 3 h prior to the testing session (Thijssen et al. 2011). Data collection was performed in a quiet, temperature-controlled room (19–23.5°C).

Upon arrival at the laboratory, participants were positioned supine with the exercising (left) arm extended 90° at heart level. Participants performed three maximal effort isometric forearm handgrip contractions using a handgrip dynamometer, each separated by 1 min of rest. The largest force was taken to represent the maximum voluntary contraction (MVC). Participants then rested for approximately 10–20 min to allow their hemodynamic parameters to return to resting levels (confirmed by brachial artery blood velocity measurement of ≤5 cm/sec). Following instrumentation, participants performed the fCF_impulse_ test.

#### Forearm critical force (fCF_impulse_) test

**Figure 2:**
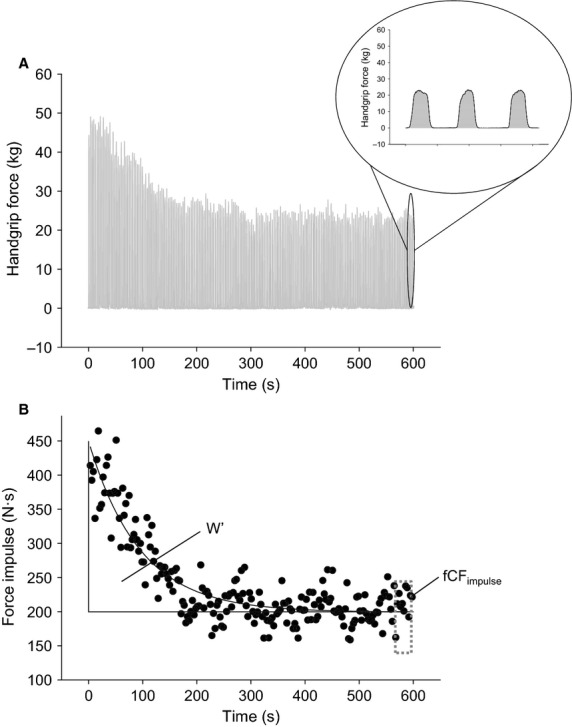
Force output during the fCF_impulse_ test for a representative participant. (A) Raw handgrip force tracing. Inset shows three contractions (shading indicates computation of the force impulse as the AUC). (B) Force impulse. Each dot represents the AUC of a single contraction. Dashed box indicates that the curve fit was used to calculate the average force impulse during the last 30 sec of the test; this value is the fCF_impulse_. The area between the fCF_impulse_ and the curve fit is the W′. fCF, forearm critical force; AUC, area under the curve.

The fCF_impulse_ test was conducted as previously described (Kellawan and Tschakovsky 2014). Briefly, after 1 min of resting baseline measures, participants performed MVCs in time with a metronome (1 sec contraction: 2 sec relaxation) for 10 min. A Powerlab oscilloscope display provided continuous force output feedback (Powerlab; AD Instruments, Colorado Springs, CO; Fig.2A), and the MVC target force was identified on the screen. Participants were not given feedback on elapsed time, but were given verbal encouragement and coaching (i.e., reminded to use the muscles of the forearm only, to relax between contractions, and to maintain the appropriate cadence).

Achievement of a valid test result is contingent on the participant having a high degree of motivation and a willingness to give maximum effort for each handgrip contraction. Even with prior test familiarization and persistent encouragement, two participants in each group performed poorly on the test (i.e., did not deliver maximal effort with each contraction, resulting in repeated cycles of declines in force production followed by recovery) and failed to reach the requisite plateau in force production. These participants were excluded from the study (data not shown; final *n = *8 per group).

### Measurements

#### Anthropometric measurements and blood sampling and analysis

Anthropometric measures (height, weight, BMI, waist circumference) and blood samples for determination of fasting plasma glucose, HbA1c, triglycerides, and total, high-density lipoprotein (HDL) and low-density lipoprotein (LDL) cholesterol were taken during the participants’ screening process for the Cardiac Rehabilitation Program at HDH. A symptom-limited graded maximal effort exercise tolerance test was performed on a treadmill (ramp protocol) and the maximum metabolic equivalents of task (METs) achieved were calculated based on the final treadmill speed and grade. Forearm volume (mL) was measured via water displacement in a custom volumeter, and forearm girth (cm) was measured with a tape measure at the point of greatest forearm circumference.

#### Mean arterial blood pressure

Mean arterial pressure (MAP) was measured continuously with finger photoplethysmography on the nonexercising arm positioned at heart level (Finometer MIDI; Finapres Medical Systems, Amsterdam, the Netherlands).

#### Brachial artery diameter

Images of the brachial artery were obtained using a 10 MHz linear Echo ultrasound probe (Vivid i2; GE Medical Systems, Waukesha, WI), operating in two-dimensional B-mode, and recorded continuously in Digital Imaging and Communications in Medicine format.

#### Brachial artery mean blood velocity

Mean blood velocity (MBV) was measured distal to the Echo probe placement with a 4 MHz Doppler ultrasound probe (Multigon 500B, Transcranial Doppler; Multigon Industries, Yonkers, NY). The corresponding voltage output was recorded continuously at 200 Hz in the data acquisition software program LabChart (ADInstruments, Colorado Springs, CO) for later analysis. To quantify absolute MBV the ultrasound probe was calibrated as follows. Briefly, the ultrasound probe was immersed in a water bath and positioned to insonate tubing of known internal diameter (angle of insonation when probe is parallel to the tube is 70°). Water with ultrasound-reflecting particles (corn starch) was pumped through the tubing at a series of known flow rates (measured volumetrically as the rate of change of fluid volume in a collecting container) representing actual mean velocities of ∼2–120 cm/sec. The voltage output was then plotted against the known mean flow velocity, giving a linear calibration slope (*r*^2^* = *0.98). This procedure was repeated with the ultrasound probe positioned at different insonation angles, manipulated to achieve 1° increments with a range of 70 ± 15°, representing the observed range in brachial artery blood vessel orientation relative to the skin. For each participant, the brachial artery was imaged at the site of Doppler probe placement to quantify the actual angle of insonation (using an on-screen protractor), and the appropriate voltage-to-velocity calibration was applied prior to data analysis. This enabled accurate absolute blood velocity measurements allowing for between-participant comparisons.

#### Handgrip force

Handgrip force was obtained using an electronic handgrip dynamometer connected to a data acquisition system (Powerlab; ADInstruments) and recorded on a personal computer (LabChart; ADInstruments).

### Data analysis

#### Forearm critical force (fCF_impulse_)

The area under the curve (AUC) of the handgrip force tracing for each forearm muscle contraction was computed (in kg·sec) and multiplied by gravitational acceleration (9.8067 m/sec^2^) to give the time–tension integral (i.e., the “force impulse”) in Newton-seconds (N·sec). The force impulse data were then fit with nonlinear regression (exponential decay; SigmaPlot 11.0; Systat Software Inc., San Jose, CA). The regression equation was used to calculate the average force impulse in the last 30 sec (∼10 contractions) of the exercise test, and this value was taken as the fCF_impulse_ (Fig.2B). Our laboratory has demonstrated excellent trial-to-trial repeatability of this test [test–retest coefficient of variation (CV) 6.7%] (Kellawan and Tschakovsky 2014).

#### Curvature constant of the force–time relationship (W′)

The W′ constitutes the maximum amount of work that can be performed above the critical force and primarily represents a fixed anaerobic energy reserve (Jones et al. 2010). The W′ was calculated as the excess impulse above fCF_impulse_ for all contractions (Fig.2B). This was derived by subtracting the calculated fCF_impulse_ from the force impulse predicted by the line of best fit for each contraction during the test, and summing these values. This is conceptually equivalent to subtracting the AUC of a horizontal line plotted at *y = fCF*_*impulse*_ from the AUC of the regression equation fit to the force impulse data (from 0 to 600 sec).

#### Mean blood velocity and mean arterial pressure

After applying the appropriate voltage-to-velocity calibration, the data collected at 200 Hz were filtered using a low-pass filter with a cut-off frequency of 0.2 Hz (lowpass.xfm, SigmaPlot 11.0, Systat Software Inc.) to eliminate higher frequency noise (i.e., higher frequencies associated with muscle contraction and cardiac cycle) (Ferreira et al. 2006). The data were then resampled at 10 Hz (i.e., from the 200 Hz data set, every 20th datum was retrieved). Resampling was necessary to reduce the overall number of data points (from ∼132,000 to 6600) in order to fit the data within the constraints of the software fitting algorithm capacity. Parameter estimates have been shown to be unaffected by resampling at 10 Hz (Ferreira et al. 2006). MAP data were similarly sampled at 200 Hz, filtered, and resampled.

#### Brachial artery diameter

Vessel diameter was quantified at 30-sec intervals using automated wall tracking as previously described (Pyke et al. 2008). Diameter data were then plotted over time and an exponential or sigmoidal line of best fit was determined, depending on the type of diameter response profile observed (SigmaPlot 11.0, Systat Software Inc.). Using the curve fit equation, diameter values were calculated for each time point at which blood velocity values were obtained. This approach minimizes the effect of random diameter measurement error on calculated blood flow.

#### Forearm blood flow (FBF), forearm vascular conductance (FVK), and MAP

Forearm blood flow was used as a surrogate for oxygen delivery and was calculated according to the formula:

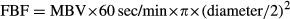
1where the FBF is in mL/min, the MBV is in cm/sec, and brachial artery diameter is in cm. Intravenous catheterization and measurements of blood oxygen content (CaO_2_) were not employed in the present study to calculate oxygen delivery directly, since our laboratory previously demonstrated that changes in FBF accounted for almost all of the between-participant differences in oxygen delivery in fCF_impulse_ tests (forward stepwise multiple regression was used to evaluate the independent contributions of FBF and CaO_2_ to oxygen delivery, and the change in *r*^2^ by their addition to the model was 0.944 and 0.0515 respectively; total *r*^2^* = *0.996, *P *< 0.001) (Kellawan et al. 2014). FVK was calculated as:

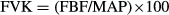
2where FVK is in mL/min/100 mmHg such that the values for FVK are quantitatively similar to those for FBF.

Data were removed where measurement error occurred (due to compromised signal). FBF, FVK, and MAP data were curve fit with two-component exponential nonlinear regression (SigmaPlot 11.0; Systat Software Inc.). For one participant in the T2D group, a one-component exponential curve was fit to the MAP data, and since the FBF and FVK showed a visible decline after the initial two-component increase, this part of the data was fit separately with linear regression. The curve fit equations were used to calculate the average FBF, FVK, and MAP at rest (“baseline”; 1 min average) and in the last 30 sec of the test (i.e., “steady state” hemodynamic values at the time of fCF_impulse_), and the change from baseline to steady state was calculated (i.e., ΔFBF, ΔFVK, ΔMAP). The AUC of the FBF curve fit was computed as a measure of the total oxygen delivered during the fCF_impulse_ test (including the resting baseline).

#### Contributions of ΔFVK and ΔMAP to ΔFBF

To determine how the increases in FBF (ΔFBF) during the fCF_impulse_ tests were achieved (i.e., from baseline to steady state), the proportional contributions of changes in FVK and MAP were calculated as follows:

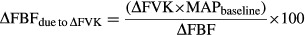
3 and

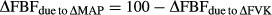
4

In other words, we calculated how much FBF would have changed if only FVK increased (assuming MAP was constant) as a proportion of the actual increase in FBF, with the remainder of the change in FBF being attributed to the increase in MAP.

#### Vasodilatory capacity

In addition, to quantify the vasodilatory “capacity” (i.e., independent of the impedance effect of muscle contraction and/or the potential immediate muscle relaxation-induced negative venous pressure), we examined the equivalent of a cardiac cycle in the relaxation phase between contractions that was unaffected by either muscle contraction or relaxation (FBF_relax_, FVK_relax_) (Walker et al. 2007). To quantify the impact of muscle contraction, we also assessed the FBF and FVK during the contraction phase of the duty cycle (FBF_contract_, FVK_contract_). These assessments were done during the final 30 sec of the test (i.e., at the time of fCF_impulse_).

### Statistical analysis

Independent *t*-tests were used to compare the effect of group (T2D, control) on participant characteristics. Data for ΔFBF, FBF_relax_, ΔFVK, and FVK_relax_ were log-transformed (natural log); this was: (1) since the data were bound by zero, (2) since the data were positively skewed, and (3) to allow a multiplicative interpretation. Independent *t*-tests were performed to compare the effect of group on the primary [fCF_impulse_; ΔFBF and FBF_relax_ (raw and log-transformed)] and secondary [total FBF; ΔFVK and FVK_relax_ (raw and log-transformed); FBF_contract_, FVK_contract_; ΔMAP; ΔFBF_due to ΔFVK_ and ΔFBF_due to ΔMAP_; and W′] outcome variables. Transforming the data did not change the statistical interpretation; raw (nontransformed) data are shown in figures for ease of interpretation with associated *P* values from the transformed data statistical analysis. The reverse of the log transformation was applied (exponentiation) to show the mean fold-difference between groups. Bootstrapping (10,000 bootstrap samples) was used to obtain valid 95% confidence intervals (CI; bias corrected accelerated) without relying on the assumption of normality. Linear regression was used to assess the relationships between each of the following and fCF_impulse_: FBF (during the last 30 sec of the fCF_impulse_ test), ΔFBF, FBF_relax_, and total FBF. Data for one participant in the T2D group was removed from comparisons involving fCF_impulse_ (between groups and in regression analysis) since: (1) the fCF_impulse_ had a *z*-score >2, (2) in fCF_impulse_ versus FBF regressions the standardized residual was >2, and (3) most importantly, an fCF_impulse_ of this magnitude would be highly unlikely given the participant’s small FBF magnitude and the known dependency of fCF_impulse_ on oxygen delivery (Kellawan 2013). This participant’s quantified fCF_impulse_ therefore likely represents the result of a handgrip position that altered the mechanical advantage (such that the measured contraction force was achieved with lower actual muscle contraction force) (Kellawan and Tschakovsky 2014), and thus this participant’s data was excluded from statistical comparisons and is not included in the group mean values (but is still plotted with distinct data points). Statistical significance was set at *P *< 0.05, and all statistics were calculated using IBM® SPSS® Statistics (version 22; SPSS Inc., Chicago, IL). All data are expressed as means ± SD.

## Results

### Participant characteristics

Participant characteristics are shown in Table1. The T2D and control groups did not differ with respect to age, height, weight, BMI, waist circumference, fasting blood lipids, METs achieved on a graded treadmill exercise test, MVC, forearm volume and circumference, and number of non-T2D medications (all *P *> 0.05). As expected, the T2D group had greater fasting plasma glucose (*P = *0.001) and HbA1c levels (*P = *0.009) versus control.

### fCF_impulse_ and W′

**Figure 3:**
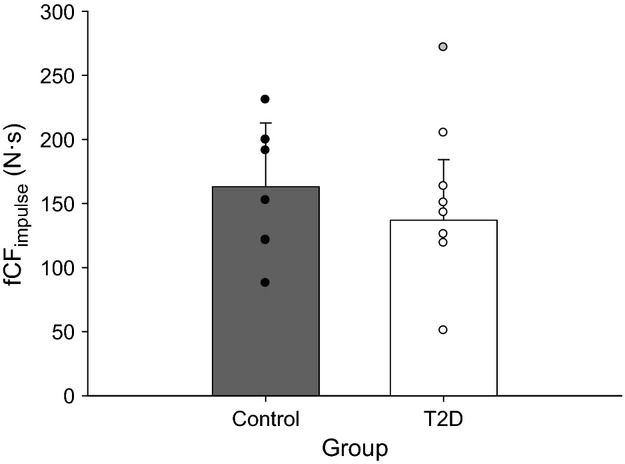
Forearm critical force. Data are means (excluding outlier in T2D group) ± SD, with individual participant points (gray point = outlier excluded from analysis).

Force output for a representative participant is shown in Figure2. fCF_impulse_ was not significantly different between groups (*P = *0.317; mean difference 26.2 N·sec, 95% CI: −16.8 to 69.9 N·sec) (Fig.3). W′ was not different between T2D and control groups (5583.4 ± 3588.3 N·sec vs. 4848.8 ± 2420.0 N·sec respectively; *P = *0.639).

### Hemodynamic parameters – FBF, FVK, MAP

**Figure 4:**
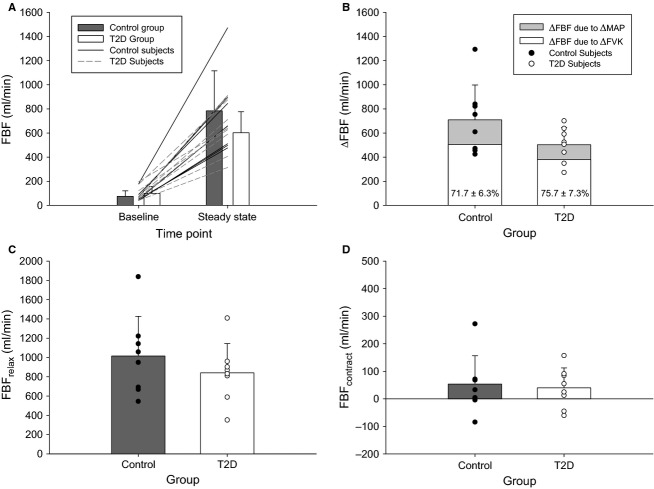
Forearm blood flow. (A) Absolute FBF at rest and during the last 30 sec of the fCF_impulse_ test (“steady state”) in the control (gray bars) and T2D (white bars) groups. Lines represent individual participant data (solid = control participants; dashed = T2D participants). (B) Change in FBF from resting baseline to the last 30 sec of the fCF_impulse_ test. White shading represents the predicted ΔFBF if only FVK changed (MAP constant), with the remaining gray shading due to the addition of a change in MAP. Inset percent values indicate the proportional contribution of ΔFVK to ΔFBF. (C) Absolute FBF_relax_ and (D) FBF_contract_ during the last 30 sec of the fCF_impulse_ test. Data are means ± SD; closed symbols = control group, open symbols = T2D group. FBF, forearm blood flow; fCF, forearm critical force; FVK, forearm vascular conductance; MAP, mean arterial pressure.

**Figure 5:**
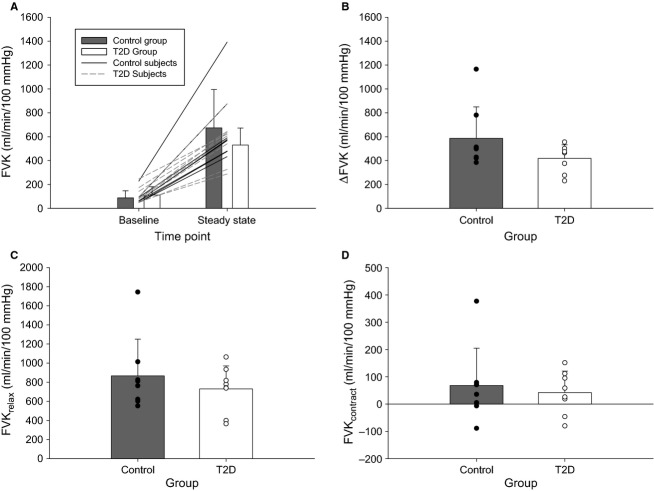
Forearm vascular conductance. (A) Absolute FVK at rest and during the last 30 sec of the fCF_impulse_ test (“steady state”) in the control (gray bars) and T2D (white bars) groups. Lines represent individual participant data (solid = control participants; dashed = T2D participants). (B) Change in FVK from resting baseline to the last 30 sec of the fCF_impulse_ test. (C) Absolute FVK_relax_ and (D) FVK_contract_ during the last 30 sec of the fCF_impulse_ test. Data are means ± SD; closed symbols = control group, open symbols = T2D group. FVK, forearm vascular conductance; fCF, forearm critical force.

No hemodynamic parameters differed between groups at baseline (for FBF, FVK, and MAP: *P = *0.359, *P = *0.460, *P = *0.542; Figs.4A, 5A).

#### Forearm blood flow

The increase in FBF from baseline to during fCF_impulse_ (ΔFBF) was not significantly different between groups (*P = *0.092) although there was considerable variability in responses (Fig.4). On average, the ΔFBF in the T2D group was 0.727 times that in the control group (95% CI: 0.511–1.02 times). The ΔFBF was achieved primarily via an increase in FVK; overall the proportional contributions of ΔFVK and ΔMAP to ΔFBF were 73.7 ± 6.9% and 26.3 ± 1.7%, respectively, with no differences between groups (both *P = *0.256; Fig.4B).

Similarly, the absolute FBF during the relaxation phase of the duty cycle (FBF_relax_) was not significantly different between groups (*P = *0.368; mean fold-difference 0.831; 95% CI for fold-difference between groups 0.553–1.212), nor was FBF during the contraction phase (FBF_contract_; *P = *0.647; Fig.4C and D).

Total FBF during the test was not different between groups (5629.4 ± 2148.0 mL vs. 6873.77 ± 3068.1 mL for T2D vs. control; *P = *0.363).

#### Forearm vascular conductance

The ΔFVK was not significantly different between groups (*P = *0.095; mean fold-difference between groups 0.735; 95% CI: 0.513–0.994; Fig.5B). FVK_relax_ was not different between groups (*P = *0.399), nor was FVK_contract_ (*P = *0.762; Fig.5C and D).

#### Mean arterial pressure

On average, MAP was 89.0 ± 10.6 mmHg at baseline (90.7 ± 8.6 mmHg vs. 87.4 ± 12.6 mmHg for T2D vs. Control; *P = *0.542) and increased to 116.9 ± 18.6 mmHg at the time of fCF_impulse_ (115.5 ± 17.4 vs. 118.3 ± 20.8 for T2D vs. Control) and the increase from baseline was not different between groups (*P = *0.259).

### Linear regressions

**Figure 6:**
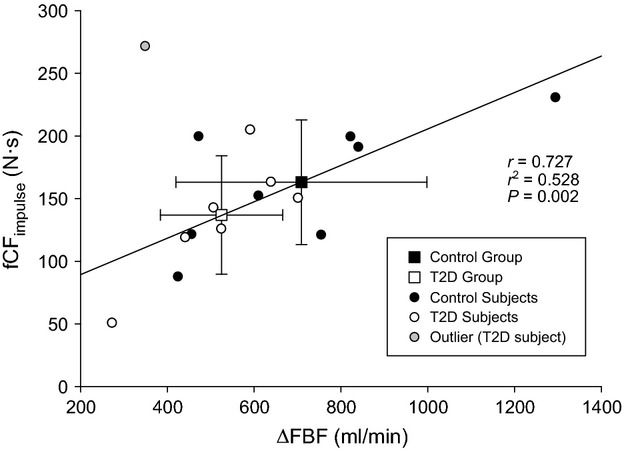
Relationship between the increase in FBF during the fCF_impulse_ test (ΔFBF) and fCF_impulse_. Squares are group means ± SD; circles are individual participant points (closed symbols = control; open symbols = T2D; gray data point = outlier excluded from analysis). FBF, forearm blood flow; fCF, forearm critical force.

Absolute FBF at fCF_impulse_, ΔFBF, FBF_relax_, and total FBF were significantly related to fCF_impulse_ (all *P *< 0.05; Fig.6, Table2).

**Table 2:** Linear regressions: relationship between the following variables and fCF_impulse_

Variable	*r*	*r* ^2^	*P*
Absolute FBF at steady state (mL/min)	0.690	0.476	0.004
ΔFBF (mL/min)	0.727	0.528	0.002
FBF_relax_ (mL/min)	0.664	0.441	0.007
Total FBF (mL/min)	0.663	0.440	0.007

*n* = 15 (outlier excluded from regression analysis).

fCF, forearm critical force; FBF, forearm blood flow.

## Discussion

The objective of this study was to test the hypothesis that T2D, when present in an ecologically valid constellation of comorbidities and medications, compromises small muscle mass exercise tolerance and that this is related to blunted exercising muscle blood flow. Contrary to our hypothesis, the major findings of this study are: (1) small muscle mass exercise tolerance (fCF_impulse_) was not impaired in T2D relative to matched controls, and (2) there was no difference in forearm muscle blood flow (or its determinants, FVK and MAP) during exercise at fCF_impulse_ between T2D and control groups, with considerable variability between individuals. These data also provide evidence for a strong relationship between oxygen delivery and exercise tolerance (fCF_impulse_).

### Small muscle mass exercise tolerance (fCF_impulse_) in T2D

This is the first study, to the best of our knowledge, to examine the impact of T2D on small muscle mass exercise tolerance when accompanied by the typical constellation of comorbidities and associated medications in this population. Contrary to our hypothesis, fCF_impulse_ was not different between persons with T2D and matched controls. In addition, W′ (the curvature constant of the force–time relationship) was not different between groups, indicating that the finite amount of work that could be performed above fCF_impulse_ was also not affected by T2D. These findings have functional significance since small muscle mass exercise tolerance is central to conducting activities of daily living.

Evidence for impaired exercise tolerance in T2D comes primarily from observations of reduced peak oxygen consumption capacity [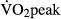; ∼20% reduction versus controls matched for age, BMI, and *self-reported* physical activity level (Regensteiner et al. 1995, 1998; Baldi et al. 2003; Lalande et al. 2008)]. However, this evidence is potentially problematic since self-reported activity has limited reliability and validity (Shephard 2003), and since overweight/obese populations are known to systematically overestimate levels of physical activity (Prince et al. 2008). Indeed, objective measures of physical activity (accelerometry) confirmed that among sedentary adults (<1 h/week of moderate intensity physical activity), those with T2D spent significantly more time being inactive and less time at all levels of intensity (light, moderate, vigorous) than those without T2D (matched for age and BMI) (O’Connor et al. 2012). Thus, it is possible that the findings of impaired peak aerobic function in T2D are due to a more sedentary lifestyle (Thomas et al. 2004; Reusch et al. 2013) versus disease-related dysfunction. In the present study, the T2D and control groups were matched for maximal METs achieved on a graded treadmill exercise test in order to remove fitness as a potential confounder. The current findings do not support the hypothesis that T2D, on top of the typical constellation of comorbidities present in this population, impairs small muscle mass exercise tolerance.

### Exercising muscle oxygen delivery in T2D – muscle blood flow

Forearm blood flow during exercise at fCF_impulse_ did not differ between T2D and control groups in the present study, and the similar FBF was achieved via similar FVK and MAP responses (Figs.4, 5). This was confirmed by examining FBF and FVK in terms of the mean response, as well as specific to relaxation and contraction phases of the duty cycle (the relaxation phase being an indicator of the vasodilatory response, and the contraction phase being a gauge of the mechanical impedance to flow during the maximal effort test), and the total FBF across the test (AUC).

These results were unexpected given the evidence for impaired vasodilatory mechanisms in T2D (Hogikyan et al. 1999; James et al. 2004; Sprague et al. 2006; Montero et al. 2013) and the findings of two recent studies which formed the basis for our hypothesis (Kingwell et al. 2003; Lalande et al. 2008). First, Kingwell et al. (2003) observed reduced steady state leg blood flow (LBF) in persons with T2D versus Controls (matched for age, 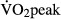, and body weight) during moderate-intensity exercise (25-min supine cycling at 60% 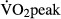). Similarly, Lalande et al. (2008) demonstrated that steady state LBF, albeit indexed to lean thigh mass, was significantly lower in T2D participants versus healthy controls (matched for age, BMI, and fat-free mass) during low-intensity leg-kicking exercise. In contrast to these previous investigations of exercising leg muscle blood flow, however, the present study is the first to evaluate the impact of T2D on exercising muscle blood flow and exercise tolerance within the typical clustering of comorbidities and medications in this population, and it is within this important context that we suggest possible explanations for the current findings.

#### One or a combination of the other comorbidities may have equally impaired exercising muscle blood flow in both groups

First, it is possible that one or more comorbidities contributed to impaired FBF such that both groups were impacted to the same extent independent of T2D. For instance, chronic hypertension is associated with vascular remodeling (and increased media/lumen ratio), increased artery stiffness (Intengan and Schiffrin 2001) and damaged capillaries (Levy et al. 2001), while dyslipidemia is known to result in heightened inflammation, impaired endothelial function (Chehade et al. 2013), and atherosclerosis (Malloy and Kane 2004), all of which might be expected to impact exercising muscle blood flow. Similarly, obesity could compromise exercising muscle blood flow via myogenic, endothelium-dependent, and metabolic control mechanisms (Hodnett and Hester 2007), although impairment is not always demonstrated (Limberg et al. 1985).

Therefore, it may be that T2D has no independent effect, or that it has no additional effect when superimposed on the comorbidities that usually accompany the disease (i.e., due to a “floor effect”). This hypothesis is supported by previous studies in which an impairment in persons with T2D was observed only when compared with lean (but not weight-matched) controls (Regensteiner et al. 1998; Sanchez et al. 2011). In previous investigations in which control participants were not matched for these characteristics (Womack et al. 2009; Joshi et al. 2010), the conclusion of an impact of T2D per se may have been confounded by the presence of clinical sequelae (i.e., spurious associations due to comorbid conditions but falsely attributed to T2D). While T2D may have an independent effect when unaccompanied by comorbidities (Bauer et al. 2007; Lalande et al. 2008), it should be recognized that these otherwise healthy individuals represent a minority of those with T2D. These possibilities do not alter the interpretation of the present study however, which is that in the “average” person with T2D, exercising FBF is not reduced relative to an otherwise similar individual in the absence of T2D.

#### Regular medications may moderate the impact of T2D on exercising muscle blood flow

We also elected to test participants without disruption of their regular medications. As previously mentioned, the important rationale for this was that the majority of persons with T2D take antidiabetic, antihypertensive, and antihyperlipidemic agents (Krentz and Bailey 2005; Savoia and Schiffrin 2007), making such participants representative of the general T2D population and improving the ecological and external validity of our findings.

For instance, up to 80% of persons with T2D also have hypertension (Savoia and Schiffrin 2007), and of those more than 65% require ≥2 medications to achieve the target blood pressure of <130/85 mmHg, with angiotensin-converting enzyme (ACE) inhibitors being the first prescribed (Sowers et al. 2001). Even in persons with T2D and “normal” blood pressure, ACE inhibitors are often prescribed since they are known to reduce the progression of renal (Ravid et al. 1996) and cardiovascular (Yusuf et al. 2000) disease in this population (Sowers et al. 2001). Similarly, statins are prescribed for treatment of dyslipidemia. Based on their mechanisms of action, both of these classes of medications might be anticipated to influence exercising muscle blood flow, and indeed this has been demonstrated for antihypertensives (Yang and Terjung 1985; Drexler et al. 1989; Cowley et al. 1993). Importantly, these medications were well-matched between groups in the present study (Table1) and thus any potential impacts would be exerted equally on both groups.

In contrast, in the T2D group there was the additional potential impact of T2D-specific medications. Antidiabetic agents are known to improve endothelial function (Mather et al. 2001) and there is some (limited) evidence that they may increase exercising muscle blood flow (Hallsten et al. 2002). The absence of antidiabetic medications in the studies mentioned earlier could potentially explain the findings of impaired muscle blood flow in T2D, since Lalande et al. (2008) enrolled only medication-free participants, and Kingwell et al. (2003) included only two T2D participants who took antidiabetic agents but completed testing after a 24 h drug-free period.

In the present study, seven T2D participants were taking Metformin, with one also taking insulin. While these medications may therefore have contributed to the conserved FBF responses, we cannot estimate the magnitude of their effect (if any) (Baldi et al. 2003). In addition, others have observed no impact of T2D on steady-state muscle blood flow when medications were held for 48 h before testing (Martin et al. 1995), or impaired muscle blood flow when participants were tested while on their regular medications [only when HbA1c ≥8% (Joshi et al. 2010)]. Thus, differences in medication status cannot fully account for differences in exercising muscle blood flow responses in T2D across studies.

#### The impact of T2D may depend on the active muscle mass and/or the specific vascular bed

It is possible that there is an impairment in muscle blood flow during leg but not arm exercise in persons with T2D, as a result of differences in active muscle mass and/or possible differences in muscle blood flow regulatory mechanisms across vascular beds (Newcomer et al. 2004; Richardson et al. 2006). Importantly, however, we (unpubl. obs.) and others (Martin et al. 1995; MacAnaney et al. 2011; Slade et al. 2011; Thaning et al. 2011) have found no impairment to steady-state LBF during low- to moderate intensity leg exercise (comparable to that in Kingwell et al. 2003; Lalande et al. 2008) in persons with T2D versus controls. This suggests that differences in muscle mass and/or vascular beds do not present a straightforward explanation for disparate findings across studies.

#### There may be different cohorts within persons with T2D [such that (dys)function exists along a continuum]

Finally, we propose that some persons may be more susceptible to a negative impact of T2D than others, such that some exhibit diabetes-evoked impairment and some do not. This hypothesis is supported by the substantial interindividual variability in FBF responses in the present study. In the T2D group, the range in ΔFBF responses from baseline to during exercise at fCF_impulse_ was ∼270 to 700 mL/min (i.e., the participant with the largest ΔFBF had an increase in FBF more than double that of the individual with the lowest ΔFBF, for a difference of 430 mL/min; see Fig.4B). This between-participant range in responses is considerable and would be expected to translate to functional impairment. (Indeed, the difference in fCF_impulse_ between these two participants was ∼100 N·sec.) Thus, while at the group level impairment was not observed, the large demonstrated variability in responses suggests that impaired and unimpaired cohorts may exist within this population, and this could explain some of the inconsistency in the literature [i.e., some have found impaired exercising muscle blood flow in persons with T2D (Kingwell et al. 2003; Lalande et al. 2008; Young et al. 1991), while others have not (Martin et al. 1995; Behnke et al. 2002; Padilla et al. 2007; Copp et al. 2010; Thaning et al. 2011)]. Note however that there was also large variability within the control group, and thus the interindividual differences may be moderated by factors other than T2D specifically. Future work will be required to identify whether different phenotypes exist and the specific conditions in which impairment does versus does not manifest.

### Implications of preserved exercising FBF

Type 2 diabetes is characterized by both macro- and microvessel endothelial and smooth muscle dysfunction (Montero et al. 2013), impaired release of ATP from red blood cells (Sprague et al. 2006), reduced bioavailability of nitric oxide (James et al. 2004), and potentially heightened sympathetic nervous system activity (Hogikyan et al. 1999), all of which would be anticipated to reduce exercising muscle blood flow. Given this evidence for vascular dysfunction in persons with T2D, the present finding of preserved FBF during exercise at fCF_impulse_ was unexpected. It is known that there is considerable redundancy in mechanisms controlling exercise hyperemia (Laughlin and Korzick 2001; Joyner and Wilkins 2007), and the present results suggest that compensatory responses may function to defend exercising muscle oxygen delivery in T2D.

### The relationship between oxygen delivery and exercise tolerance

In the present study, a strong positive relationship was observed between FBF (whether expressed as absolute FBF, ΔFBF, FBF_relax_, or total FBF) and exercise tolerance (fCF_impulse_) (Fig.6, Table2). This is in agreement with previous observations in young, healthy participants (Kellawan et al. 2014) and suggests that differences in FBF (as determined by vasodilatory and pressor responses) may be an underlying mechanism explaining the interindividual differences in exercise tolerance.

### Limitations

In this study, statistical analysis of the increase in blood flow and vasodilation from rest for the T2D group versus the control group resulted in *P* values of *P = *0.092 and *P = *0.095, respectively. This raises the possibility of a type II error (i.e., failing to detect an effect in the sample that truly exists in the population), due to the limitation of a small sample size. However, additional analyses support a conclusion of no difference between groups, or at most, a clinically insignificant difference.

First, when measuring the FBF and FVK during relaxation phases between contractions (i.e., unconfounded by the potential impact of contraction-induced movement artifact on the brachial artery blood velocity measures), the *P* values for between-group comparisons were large (FBF_relax_, *P = *0.368; FVK_relax_, *P = *0.399), signifying a highly improbable difference between groups. This was also the case for total FBF (*P = *0.363). Second, bootstrapping analysis achieved valid confidence intervals of the fold-difference in responses between T2D and control groups, and the confidence interval for ΔFBF crossed 1 (i.e., no difference between groups). Third, individual variation in the increase in FBF was a strong predictor of individual variation in fCF_impulse_ (*r*^2^* = *0.528, *P = *0.002 for ΔFBF vs. fCF_impulse_), yet there was no difference between groups in fCF_impulse_ (*P = *0.317), meaning that if there was an effect of T2D on the increase in FBF that we were unable to detect, it was so small as to have no effect on fCF_impulse_. Finally, post hoc power analysis indicates that to detect between group differences in ΔFBF, assuming that additional subjects would have populated the data distribution normally toward the current outlier in the control group, would have required 33 subjects per group. Based on a data distribution with this outlier removed, 40 subjects per group would have been required. These sample sizes are much larger than the sample size typically employed in studies of this nature (i.e., a sample size of 8–10 per group is standard) (Martin et al. 1995; Neder et al. 2000; Kingwell et al. 2003; Lalande et al. 2008).

It must also be acknowledged that findings cannot be generalized to the minority of T2D patients who do not have comorbidities or who are not taking antihypertensive, antihyperlipidemic, or antidiabetic medications, or to women since there is some evidence that their exercise tolerance may be disproportionately reduced (Ades et al. 2006; Ribisl et al. 2007).

We cannot exclude the possibility that some T2D patients may have declined to participate in the study on the basis of reduced exercise capacity (Sanchez et al. 2011), thereby biasing our study sample in that it would systematically underestimate the true level of exercise intolerance experienced in the general T2D population. This seems unlikely however since the number of participants who voluntarily excluded themselves was greater in the control group (*n = *13) than the T2D group (*n = *7) (Fig.1). Similarly, the four patients who had invalid fCF_impulse_ tests and were excluded from analysis may have had reduced exercise capacity (i.e., leading to their poor performance on the test). However, since there were two participants with invalid tests per group, this is unlikely to represent any systematic effect of T2D.

The use of ultrasound quantifies brachial artery blood flow, however, it may fail to detect microvascular pathology. For instance, Womack et al. observed an impairment in microvascular perfusion that was unaccompanied by a reduction in bulk flow during low- and high-intensity handgrip exercise (25% and 80% MVC) in persons with T2D and microvascular complications versus healthy controls (Womack et al. 2009). While we cannot reject the possibility that an impairment in blood flow distribution occurred at the microvascular level in the present study, this seems improbable since no impact on exercise tolerance was observed.

Finally, we collected our data in the fasted state and others have shown that exercise hyperemia is attenuated in obese, insulin-resistant participants when exercise is performed in hyperinsulinemic conditions (Hallsten et al. 2003). Thus, it is possible that FBF and fCF_impulse_ may have been differentially affected in the postprandial state.

## Conclusions

The present study is the first to investigate the impact of T2D on small muscle mass exercising muscle blood flow and exercise tolerance within the typical constellation of comorbidities and medications that are present in this population. Contrary to our hypothesis, small muscle mass exercise tolerance and exercising muscle oxygen delivery were not impaired in representative persons with T2D (relative to controls matched for age, aerobic fitness, BMI, comorbidities, and non-T2D medications). This suggests that localized peripheral vascular control impairment does not contribute to reduced whole-body exercise tolerance in this population. There was a strong positive relationship between FBF and fCF_impulse_ suggesting that interindividual differences in exercising muscle oxygen delivery (as determined by the magnitude of the vasodilatory and pressor responses) are responsible for differences in exercise tolerance. Given the large interindividual variability in exercise hyperemia and exercise tolerance responses, future studies are warranted to more definitively establish the presence or absence of a T2D-specific impairment, and to determine whether these results can be generalized to women.
